# Delayed cerebellar ataxia induced by *Plasmodium falciparum* malaria: A rare complication

**DOI:** 10.1002/ccr3.8053

**Published:** 2023-10-20

**Authors:** Emmanuel Edwar Siddig, Sarah Misbah El‐Sadig, Hala Fathi Eltigani, Ahmed Mudawi Musa, Nouh Saad Mohamed, Ayman Ahmed

**Affiliations:** ^1^ Faculty of Medical Laboratory Sciences University of Khartoum Khartoum Sudan; ^2^ Faculty of Medicine University of Khartoum Khartoum Sudan; ^3^ Molecular Biology Unit Sirius Training and Research Centre Khartoum Sudan; ^4^ Institute of Endemic Disease University of Khartoum Khartoum Sudan; ^5^ Swiss Tropical and Public Health Institute (Swiss TPH) Allschwil Switzerland; ^6^ University of Basel Basel Switzerland

**Keywords:** critical care medicine, infectious diseases, neurology

## Abstract

**Key Clinical Message:**

In endemic areas, malaria‐induced cerebellar ataxia should be suspected in patients presenting with neurological disorders including slurred speech, tremors, and a sense of imbalance and dizziness while walking. Healthcare providers should be aware to properly investigate and early detect and manage infections associated with the development of cerebellar ataxia to improve the case management and clinical outcome cost‐effectively.

**Abstract:**

Here, we report the clinical manifestations, investigations, and outcomes of a patient developed delayed cerebellar ataxia following a malaria infection: an unusual complication of the disease. This report highlights the diagnostic challenges in a country endemic with several infectious diseases, yet it has a limited diagnostic and surveillance capacity.

## 1 INTRODUCTION

Cerebellar ataxia (CA) is a neurological condition characterized by a loss of coordination due to dysfunction in the cerebellum, a part of the brain that is responsible for the motor control.^1^ Various underlying conditions can be involved in the development of CA, including stroke, tumors, exposure to toxins such as alcohol abuse, and infections with several different diseases like arboviral diseases and malaria.^2^, ^3^ It is important to note that Sudan is endemic with several infectious diseases that are involved in the development of CA, including COVID‐19,^4^, ^5^ arboviruses such as Chikungunya,^6^ Crimean–Congo hemorrhagic fever (CCHF),^7^ dengue,^2^ Rift Valley fever,^8^ West Nile virus,^9^ Zika,^10^ and Yellow fever^11^ as well as emerging infectious diseases like schistosomiasis, TB, and different fungal infection.^12^ Furthermore, malaria is hyperendemic in Sudan^13^ and in addition to it is major role in the development of CA, it involves in several other neurological disorders.^14^

In 1984, Senanayake et al reported the occurrence of delayed cerebellar ataxia after malaria in Sri Lanka. This condition is characterized by acute and transient symptoms, manifesting either after the resolution of the fever phase of malaria or as an adverse effect of antimalarial medications.^15^ In this report, we discuss a case of delayed cerebellar ataxia as a complication of a *P. falciparum* malarial infection and highlight the importance of considering such condition in the differential diagnosis, especially in malaria‐endemic areas.

## 2 CASE PRESENTATION

A 49‐year‐old female presented to a hospital in Khartoum state, central Sudan with slurred speech, tremors affecting her upper limbs, and a sense of imbalance and dizziness while walking that lasted for 5 days. Twelve days prior, she reported a 3‐day history of fever with chills for which she took paracetamol tables 500 mg four time per day. The patient had no history of a rash, common cold, nor headache as well as no neck pain, sensory or motor deficit. Also, she has no history of bulbar symptoms, vomiting, recent vaccination, or alcohol abuse as well as no smoking, joint pains, or fluctuation in body weight. Before admission, she was not diagnosed with malaria, nor did she receive any antimalarial therapy.

On the clinical examination, the patient was conscious and oriented to time, place, and person. She had a normal pulse rate (76/min), respiratory rate (17/min), blood pressure (90/50), and temperature (39°C). Neurological examination showed no evidence of meningism. However, the patient had an ataxic gait, dysarthria, tremor of the upper limbs, dysdiadochokinesia, and hypotonia, but no bradykinesia, rigidity, and nystagmus. Furthermore, she had normal sensation. Magnetic resonance imaging (MRI) of the brain was done in order to rule out the possibility of post‐infectious cerebellitis, and it showed normal brain structures, ventricular system, no evidence of hemorrhage or infarct, and no midline shift. An electroencephalogram was done, and it was also normal.

A blood sample was collected from the patient and sent to the laboratory for routine investigations. Further investigations revealed microcytic hypochromic anemia and confirmed the presence of *Plasmodium falciparum* gametocytes in the peripheral blood smear. Viral screening for Epstein–Barr virus (EBV), cytomegalovirus (CMV), major endemic arboviruses, human immunodeficiency (HIV), and Hepatitis viruses were negative. Liver function test revealed a serum bilirubin 0.7 mg/dL, total protein 7.6 g/dL, serum albumin 5.8 g/dL, alkaline phosphatase 79 U/L, aspartate aminotransferase (AST) 21 U/L, and alanine aminotransferase (ALT) 26 U/L. Renal functional test showed a normal value of urea in blood (27 mg/dL) and serum creatinine (0.71 mg/dL). Complete blood count examination showed leucocytosis (12.3 × 10^3^), hemoglobin 11.0 g/dL, and platelet count 149 × 10^3^. A sputum sample was collected and was negative for acid‐fast bacilli.

The patient received artemether/lumefantrine four tablets (20 mg artemether; 120 mg lumefantrine per tablet) orally (PO) as an initial dose, followed by four tablets P.O. 8 h later, then four tablets P.O. twice daily (morning and evening) for 2 days for a total course of 24 tablets. She responded well after 3 days. The patient was discharged 21 days after admission. She has recovered and regained a normal health status.

## 3 DISCUSSION

In this communication, we report a case of delayed cerebellar ataxia due to *P. falciparum* infection with the onset of slurred speech, bilateral tremors affecting both upper limbs, and an unsteady gait during walking. Cerebellar involvement of *P. falciparum* malaria can occur during the acute stage of fever, as a consequence of cerebral malaria, as a delayed cerebellar ataxia (DCA), or as a side effect of antimalarial therapy.^16^, ^17^, ^18^, ^19^, ^20^, ^21^ This case of DCA is induced by malaria infection has occurred in an area hyperendemic with malaria, namely Khartoum state in central Sudan. The development of DCA in this case could be mainly attributed to the lack of detecting and treating the malaria infection during the initial presentation of the patient at the outpatient clinic. This delay in reaching a final accurate diagnosis is of high risk particularly in settings like Sudan that are endemic with several life‐threatening infections like hemorrhagic fevers. Such delay commonly leads to the development of disease severe sequelae and complication such as neurological syndromes including Guillain–Barre syndrome (GBS) and CA.^4^, ^22^

Cerebellar ataxia can be caused by many conditions, including alcohol abuse, stroke, brain degeneration, multiple sclerosis, drugs, genetic and autoimmune diseases, as well as several infectious diseases, and even vitamin E deficiency.^16^, ^17^ Malaria is one of the leading causes for the development of CA.^18^, ^19^, ^20^, ^21^, ^22^ Malaria in humans is commonly caused by one of five species of plasmodium, and *P. falciparum* species causes the majority of malaria infections in Africa including Sudan and it is particularly associated with the development of neurological complications.^23^

Acute cerebellar ataxia can be caused by a wide range of infections including viral, bacterial, fungal, and parasitic infections. Interestingly, in our reported case, there was no clinical or molecular evidence of any other infection in addition to *P. falciparum*. Co‐infection with main CA‐associated viral infections that are prevalent in the country was excluded by screening the blood sample serologically and molecularly. Additionally, in our reported case, hyperpyrexia is unlikely to cause cerebellar ataxia as our patient developed DCA after a febrile period. Therefore, the development of DCA can be directly attributed to the *P. falciparum* infection. The pathogenesis of DCA due to malaria infection is attributable to an immune mechanisms that include elevated levels of certain cytokines such as Interleukin (IL)‐2, IL‐6, and tumor necrosis factor alpha (TNF‐α), as these cytokines were found in the cerebrospinal fluid of patients with DCA.^24^

Therefore, in countries like Sudan that are endemic with malaria and other CA‐associated infectious diseases, it is crucial to investigate patients with cerebellar ataxia for these infections. Early diagnosis and effective case management of patients with infectious diseases is the main strategy to reduce the development and prevalence of CA in the country. Therefore, healthcare providers work in such settings should be vigilant and improve the differential diagnosis of cerebellar ataxia by taking a comprehensive medical and travel histories combined with a complete clinical examination and recommendations for robust laboratorial examinations to improve the diagnosis. Furthermore, in countries endemic with several infectious diseases with similarity in their clinical manifestations, more investment should be made on improving the diagnostic capacity. Furthermore, considering that in addition to malaria, the burden of arboviral diseases is rapidly growing in the country, it would be more cost‐effective to invest on prevention and control strategies that are effective against all vector‐borne diseases. Such strategies include improving the living‐environment and the implementation of early warning system with integrated vectors surveillance and response systems.^25^

Although malaria is hyperendemic in Sudan with *P. falciparum*, as the predominant species, yet development of neurological syndromes that are associated with malaria infection including CA are understudied. Therefore, more investment is needed to further study sequelae and severe complications that are associated with endemic diseases and their prevalence in the region. Particularly that, such studies are warrant to generate evidence to inform and guide policymaking and strategic planning and implementation of effective prevention and control measures to reduce the health and socioeconomic burden of such preventable health conditions.

## AUTHOR CONTRIBUTIONS

**Emmanuel Edwar Siddig:** Conceptualization; data curation; formal analysis; investigation; methodology; supervision; validation; visualization; writing – original draft; writing – review and editing. **Sarah Misbah El‐Sadig:** Investigation; writing – review and editing. **Hala Fathi Eltigani:** Investigation; writing – original draft; writing – review and editing. **Ahmed Mudawi Musa:** Investigation; writing – review and editing. **Nouh Saad Mohamed:** Methodology; writing – review and editing. **Ayman Ahmed:** Conceptualization; data curation; formal analysis; investigation; methodology; resources; supervision; validation; visualization; writing – original draft; writing – review and editing.

## FUNDING INFORMATION

None.

## CONFLICT OF INTEREST STATEMENT

The author reports no conflicts of interest in this work.

## CONSENT

Written informed consent was obtained from the patient to publish this report in accordance with the journal's patient consent policy.

## Data Availability

The data that support the findings of this study are available from the corresponding author upon reasonable request.
